# The Mediating Role of Optimism on the Relationship of Activities of Daily Living and Well-Being Among Older Adults

**DOI:** 10.1093/geroni/igaa057.1569

**Published:** 2020-12-16

**Authors:** Kent Jason Cheng, Darcy McMaughan, Matthew Smith

**Affiliations:** 1 Syracuse University, Syracuse, New York, United States; 2 Texas A&M University, College Station, Texas, United States

## Abstract

Limitations on activities of daily living (ADL) and instrumental activities of daily living (IADL) can be deleterious to an older person’s life satisfaction and overall feelings of wellbeing. This study explored the possible mediating role of optimism on relationship between changes in ADL/IADL and life satisfaction over time. Using 2006-2016 data from the Health and Retirement Study (n=11,869), growth curve modelling was used to account for intra-individual and inter-individual changes in life satisfaction trajectories. All models controlled for age, sex, marital status, years of education, self-rated health, labor force status, log of household income, and attrition. In the baseline model without optimism and with all controls, coefficients for ADL (Beta=-0.13, P<0.01) and IADL (Beta=-0.12, P<0.01) were negatively significantly associated with life satisfaction. When optimism was introduced to the model, coefficients for both ADL and IADL increased by 0.01 and remained statistically significant, which suggests some mediating effects. When interaction terms between ADL/IADL and optimism were introduced, coefficients for ADL and IADL became statistically insignificant. However, the interaction between ADL and optimism (Beta=-0.02, P<0.05) was negatively significantly associated with life satisfaction. Findings suggest that optimism may protect against the negative impact of ADL/ IADL on life satisfaction. While changes in physical functioning and mobility may influence mental health status (e.g., depression, feelings of isolation), such consequences are not inevitable. Efforts are needed to highlight the positive aspects of aging and opportunities for life enrichment to increase morale and optimism among older adults.

